# Investigation of the changes of biophysical/mechanical characteristics of differentiating preosteoblasts in vitro

**DOI:** 10.1186/s40824-015-0046-y

**Published:** 2015-11-10

**Authors:** Ramesh Subbiah, Muhammad Suhaeri, Mintai Peter Hwang, Woojun Kim, Kwideok Park

**Affiliations:** Center for Biomaterials, Korea Institute of Science and Technology (KIST), Hwarangno 14-gil 5, Seongbuk-gu, Seoul 136-791 Korea; Department of Biomedical Engineering, Korea University of Science and Technology (UST), Daejon, 305-350 Korea

**Keywords:** Biophysical properties, Atomic force microscopy, Preosteoblasts, Osteogenesis, Focal adhesion, Young’s modulus

## Abstract

**Background:**

Topography, stiffness, and composition of biomaterials play a crucial role in cell behaviors. In this study, we have investigated biochemical (gene markers), biophysical (roughness), and biomechanical (stiffness) changes during the osteogenic differentiation of preosteoblasts on gelatin matrices.

**Results:**

Our results demonstrate that gelatin matrices offer a favorable microenvironment for preosteoblasts as determined by focal adhesion and filopodia formation. The osteogenic differentiation potential of preosteoblasts on gelatin matrices is confirmed by qualitative (Alizarin red, von kossa staining, immunofluorescence, and gene expression) and quantitative analyses (alkaline phosphatase activity and calcium content). The biomechanical and biophysical properties of differentiating preosteoblasts are analyzed using atomic force microscopy (AFM) and micro indentation. The results show sequential and significant increases in preosteoblasts roughness and stiffness during osteogenic differentiation, both of which are directly proportional to the progress of osteogenesis. Cell proliferation, height, and spreading area seem to have no direct correlation with differentiation; however, they may be indirectly related to osteogenesis.

**Conclusions:**

The increased stiffness and roughness is attributed to the mineralized bone matrix and enhanced osteogenic extracellular matrix protein. This report indicates that biophysical and biomechanical aspects during *in vitro* cellular/extracellular changes can be used as biomarkers for the analysis of cell differentiation.

## Background

Topography, mechanical properties, and composition of extracellular matrices (ECM) play critical roles in determining cell fate [1]. Earlier study of matrix stiffness on stem cell destiny by Engler et al.[2], and a recent report of stem cell fate regulation via ECM tethering by Trappmann et al. [3] are among the notable studies in the field. Accordingly, factors influencing stem cell fate continue to be revealed; topographical features (biophysical cues) include alignment [4], symmetry [5], patterning [6], growth factors (biochemical cues) [7, 8], and stiffness (biomechanical properties) [2]. These factors alter not only the phenotype and function of cells but also the mechanical properties during the course of cell differentiation [9–11]. It is proven that the biochemical, biophysical, and biomechanical changes during stem cell differentiation are influenced by material features [10], wherein ECM, actin cytoskeleton (CSK), filopodia, and vinculin, focal adhesion (FA) molecule are reported to possess critical roles [12, 13]. Filopodias are the protruding fibrous bundles of cells that recognize topographic features of materials, while lamellipodia mediate spreading mechanism [13]. Their interplay on the organization of actin CSK and integrin activated focal adhesion kinase (FAK) accelerate stem cell differentiation via mechanotransduction [14]. Hence, integrated information of a variety of individual factors such as FA, gene markers, spreading area, height, roughness, and stiffness of cells can give a novel insight toward understanding and even predicting cell behaviors more precisely.

In addition, some changes to the biochemical and physicomechanical properties of differentiating cells have been reported [10, 11, 15, 16]. There are some techniques available to study the physicomechanical properties of cells, such as atomic force microscopy (AFM), magnetic twisting cytometry, micropipette aspiration, optical tweezers or a laser trap, shear-flow methods, and stretching devices [17]. Among the current techniques, AFM is a powerful tool that is useful in obtaining not only surface topography but specific information on cellular mechanical properties and interfacial forces, such as interaction, adhesion, and repulsive forces [18]. Not surprisingly, AFM is widely used to evaluate the biomechanical properties of various cell-derived ECM [19] and stem cell differentiation potentials [20], and to detect breast cancer [21] and even for diagnostic purpose of osteoarthritis [22]. In our previous report, we had evaluated the toxicity of nanomaterials by integrating the changes in aforementioned biochemico-physico-mechanical properties of cells [18].

In this study, we investigate the temporal changes of both biochemical and physicomechanical properties of differentiating preosteoblasts. Gelatin matrices functionalized on glass surfaces via poly (maleic anhydride-alt-1-octadecene) (POMA) was used as an osteogenesis-inducing substrate. Gelatin is chosen due to its dependable properties, such as biocompatibility, ease of modification, and its wide use in cell culture [23]. Osteogenic differentiation of preosteoblasts on gelatin matrices was characterized sequentially using immunofluorescence staining of osteogenic markers, von Kossa, Alizarin red staining, calcium content, and alkaline phosphatase (ALP) activity. AFM and micro indentation technique is effective in evaluating cellular changes i.e., topography, roughness and stiffness (Young’s modulus; *E*) of cells. Our results show that gelatin matrices provide a favorable osteogenic platform for preosteoblasts with strong FA, CSK, and enhanced filopodia formation. In addition, we hypothesize that the temporal increases in stiffness and roughness of cells are directly proportional to the progress of osteogenic differentiation, wherein, changes in cell behavior are strongly influenced by the mechanics of cells. The proliferation and height of cells is indirectly proportional to spreading area and do not have a direct role in the cellular properties changes during osteogenesis.

## Methods

### Gelatin matrix preparation

Gelatin matrix-coated glass coverslips were prepared following a slight modification of previous protocols [24, 25]. Briefly, covalently immobilized thin films of maleic anhydride copolymers and gelatin matrices were deposited on glass coverslips (Marienfeld). Glass coverslips were serially cleaned in acetone, ethanol, isopropyl alcohol, and distilled water (DW) and oxidized using a solution of hydrogen peroxide:liquid ammonia:DW (1:1:5) at 70 °C for 30 min. Afterwards, the coverslips were further aminosilanized using 20 mM (3-aminopropyl) triethoxysilane (APTES) solution for 2 h, and spin-coated using 0.16 % (w/v) POMA solution (molecular weight, 40,000 g/mol) in tetrahydrofuran to covalently bind polymer thin films (~5 nm) onto the glass. The samples were annealed at 120 °C for 2 h and then immersed in 0.5 % aqueous gelatin solution at 37 °C for 1 h, resulting in a covalent bond between gelatin matrix and polymer coated glass substrate. Finally, the samples were washed thoroughly with phosphate buffered saline (PBS) to remove unbound gelatin and stored in PBS at 4 °C for up to 48 h. All reagents and materials used were purchased from Sigma-Aldrich (USA), Alfa Aesar (UK), Daejung Chemicals & Metals Co. Ltd, and Daihan Scientific (South Korea), respectively.

### Characterization

The surface morphology and properties of gelatin matrices were characterized via scanning electron microscope (SEM) at an accelerating voltage of 20 kV (Inspect F50, FEI Company, Hillsboro, OR, USA). Surface topography was also evaluated using AFM (NanoWizard II, JPK Instruments, Berlin, Germany) equipped with an inverted optical microscope (Nikon). The root mean square (RMS) roughness (R_q_) was subsequently determined using analytical software (JPK Data Processing). Micro indentation measurements were carried out to measure the *E* of gelatin matrices using a CONT-S sphere probe (400 nm radius; Nanoworld Services GmbH, Erlangen, Germany) with a 0.4 N m^−1^ force constant. In addition, the surface wettability was investigated via contact angle measurements (GBX, France).

### Cell viability, proliferation, and Focal Adhesion (FA)

Preosteoblasts (MC3T3-E4; ATCC, Manassas, VA) were seeded at a density of 2 × 10^4^/cm^2^ onto the gelatin matrices and cultured in alpha minimum essential medium (α-MEM) supplemented with 10 % fetal bovine serum (FBS) and 1 % penicillin/streptomycin (P/S). To test cell viability, once the medium was removed in 24 h and replaced with a mixture of PBS (1 ml), 2 μM calcein AM, and 4 μM ethidium bromide, cells were incubated at RT for 15 min. As the samples were washed several times, the live and dead cells were visualized via fluorescence microscopy (CKX41-F32FL; Olympus). In addition, cell proliferation rate was measured on day 1 and 3 according to the manufacturer’s instructions (CCK-8; Dojindo, Japan), in which 10 % CCK-8 solution was added to samples, incubated at 37 °C for 2 h, and measured for absorbance at 450 nm using a Multiscan microplate reader (Thermo Scientific, Rockford, IL). Fluorescence staining of the cytoskeleton (F-actin), FA molecule (vinculin), and the cell nucleus was performed in order to examine FA assembly on gelatin matrix. Rinsed with PBS twice, cells were permeabilized with 0.1 % Triton X-100. After another washing, the cells were then incubated with 1 % BSA blocking solution at RT for 15 min, and subsequently washed with PBS (×3). Cells were then incubated with mouse monoclonal anti-vinculin at 37 °C for 1 h, washed with PBS (×3), and incubated with 10 μg/mL of fluorescein isothiocyanate (FITC)-conjugated goat anti-mouse IgG (Chemicon International, Temecula, CA) and tetramethyl rhodamine isothiocyanate (TRITC)-conjugated phalloidin at RT for 1 h. Cells were washed (×3), incubated with 4,6-diamidino-2-phenylindole (DAPI) solution for 5 min, and rinsed with PBS (×3). Stained cells were kept in PBS at 4 °C before analysis via confocal microscopy (Olympus FluoView FV1000). The FA pattern of each cell was quantified with respect to the number of FA spots, and the FA area was further analyzed using Imaris software (Bitplane). Three independent samples were examined for each group and fifteen separate regions for each sample were imaged using confocal microscopy.

### In vitro osteogenic differentiation and characterization

Preosteoblasts were seeded in 6 well plates at 2 × 10^4^ cells/cm^2^ and cultured in osteogenic medium for 2 weeks under standard cell culture conditions (humidified atmosphere with 5 % CO_2_). Osteogenic medium consists of α-MEM supplemented with 10 % FBS, 1 % P/S, 10 mM β-glycerophosphate, 0.1 μM dexamethasone, and 50 μg/mL L-ascorbic-2-phosphate. The medium was changed every 2 days and the differentiation of preosteoblasts was analyzed by examining the expression of osteogenic proteins (osteocalcin [OC], type I collagen [Col I]), measurement of alkaline phosphatase (ALP) activity (LabAssay, Wako Pure Chemicals Industries, Japan), and calcium content (QuantiChrom Calcium Assay Kit; DICA-500, BioAssay systems, USA), and by assessing mineralization via Alizarin red and von Kossa staining, respectively. ALP activity and calcium content were normalized to the amount of total protein of each sample, which was determined using a bicinchoninic acid (BCA) assay kit (Pierce). Fluorescent signals of the target proteins were detected via confocal microscopy (Olympus) while Alizarin red and von Kossa staining were imaged using SEM (Phenom G2 pro desktop, Eindhoven, Netherlands). Images were further analyzed quantitatively using image processing software (Image J, NIH).

### Biophysical and biomechanical properties of cells

Biophysical properties i.e., cell morphology, cell height, spreading area, and roughness were analyzed on days 1, 3, 7, and 14 using AFM equipped with a HYDRA2R-50NG probe in liquid contact mode with a scan size of 100×100 μm. SEM was also employed, in which cells were fixed, serially dehydrated in graded ethanol solution (50–100 %), completely dried, and sputter-coated with platinum before SEM observation. Young’s modulus (*E*) was also measured following the protocol from a previously published article [26]. Micro-indentation measurements were carried out using a sphere probe (5 μm radius SiO_2_; Au surface; Novascan) with a 0.01 N/m force constant. The ramp size and loading speed were set to 1 μm and 1 μm/s, respectively. A series of indentation forces (0.5 ~ 10 nN) were tested to calibrate the indentation depth in the range of 50–500 nm in order to minimize cell surface defects and Hertz model limitation. Tip-sample separation curves and the *E* was determined via Hertz’s contact model using JPK data processing software (JPK instruments), in which the Poisson’s ratio was set to 0.5.

### Statistical analysis

All data was obtained from three samples (*n* = 3) in each group, and three independent experiments were conducted. The data are presented as mean ± standard deviation (SD). A one-way ANOVA was performed to assess a statistical significance via software (Graphpad Prism 5) and the difference is considered significant if *p* value is less than 0.05.

## Results and discussions

Biophysical (nanotopography), biomechanical (*E*), and biochemical (growth factors) cues are vital for stem cell differentiation [2, 7, 8, 14]. Likewise, cellular behavior (height and spreading area) and its interplay with biophysical (roughness) and biomechanical (stiffness) properties are to dramatically change during the course of cell differentiation. Here, an attempt was made to investigate the relationship between cell behavior and biochemical or physicomechanical properties in a time-dependent manner during *in vitro* osteogenic differentiation of preosteoblasts.

**Fig. 1:**
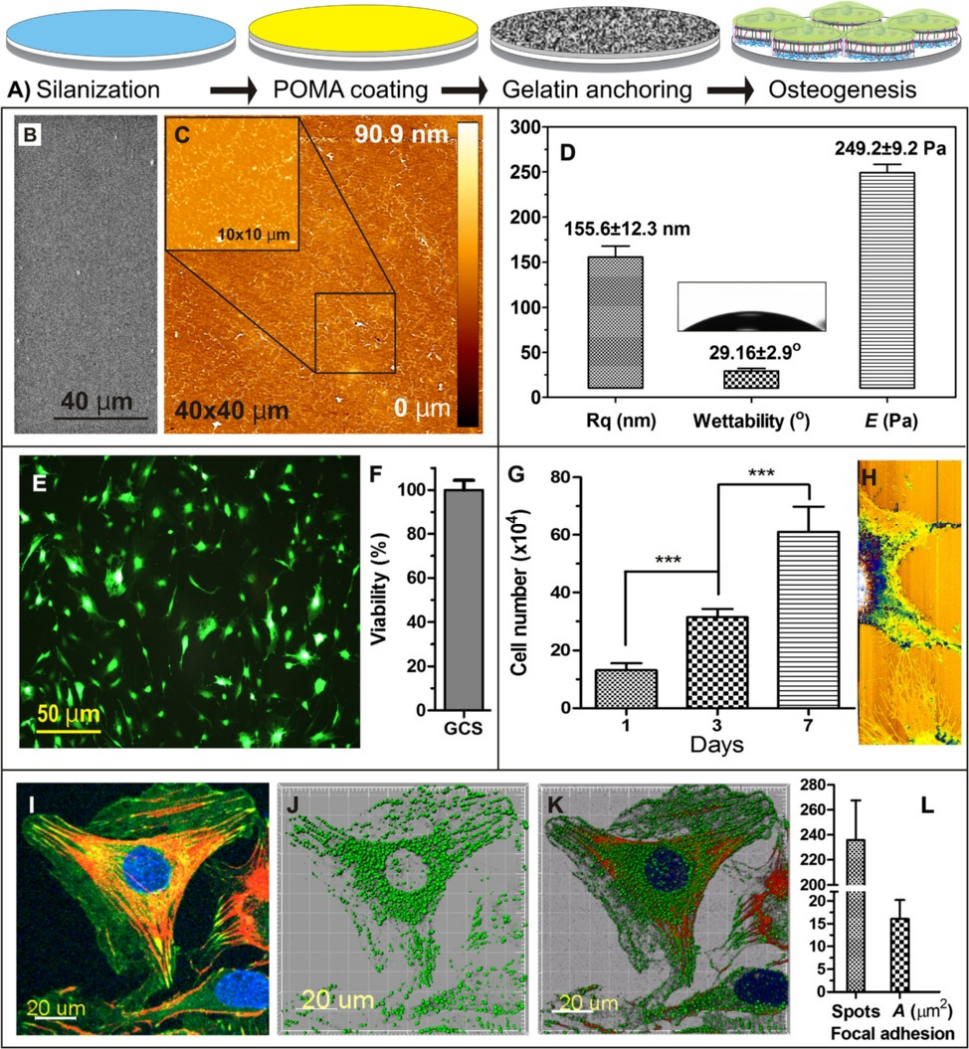
Gelatin matrices preparation, characterization and cell behavior. **a** Schematic illustration of gelatin matrix functionalization via silanization and POMA coating. **b**, **c** SEM and AFM images of gelatin matrices (*inset shows high magnification*). **d** Surface roughness (Rq), water contact angle (CA), and Young’s modulus (YM; *E*). **e**, **f**, **g**) Live/dead cell assay, viability, and preosteoblast proliferation on gelatin matrices, respectively. **h**) Live cell imaging of preosteoblasts cultured on gelatin matrices scanned in AFM liquid contact mode at day 1, wherein fiber-like structures indicate filopodia. Immunofluorescence images of **i** 2D image of cell spreading, **j** FA spots and **k** merged 3D images of preosteoblasts (green: vinculin, red: F-actin, blue: nucleus). **l**) Number of adhesion spots (#) and the average occupied area (*A*) of FA. Results are mean ± SD of one triplicate approach that is representative of three independent experiments. *** *p* < 0.001 indicates a statistically significant difference

Gelatin has been widely used in pharmaceuticals and tissue engineering applications due to its versatile properties [27]. It mimics natural extracellular microenvironment which offers biocompatibility and degradability that better suits the needs of stem cell studies, regenerative medicine, and bionanotechnology. Gelatin matrices were functionalized on glass surfaces using POMA and APTES according to the previous report [24, 25]. The schematic illustrates the covalent functionalization of gelatin matrices on POMA-coated glass coverslips (Fig. 1a). The surface morphology of gelatin matrix is visualized using AFM and SEM, respectively (Fig. 1b and c). The gelatin matrices have a wrinkled structural arrangement with a peak-to-valley depth of 70 nm. This chemistry-based surface modification evinced the formation of nanotopographic gelatin surfaces. The coating was uniform with a thickness of 200 ± 30 nm. AFM images also depicted a relatively smooth gelatin surface with an RMS Rq of 155.6 ± 18 nm (Fig. 1d). Contact angle of gelatin matrix was quite hydrophilic (29°) and *E* of gelatin matrix was ~249 Pa.

Cells on the gelatin matrix were all mostly viable after 24 h (Fig. 1e & f). Similarly, as assessed via CCK-8 assay performed at different time points (Day 1, 3, 7), a gradual increase in cell proliferation was observed (Fig. 1g). In addition, lamellipodia with protruded filopodia between neighboring cells were confirmed via live cell AFM imaging in liquid contact mode (Figs. 1h and 3e). This is a strong indication of cell migration and cell-cell interactions on the surface of gelatin matrices. Expression of focal adhesion molecule (vinculin) was screened in 24 h to understand the effect of gelatin topography on preosteoblast behavior (Fig. 1i-k). Individual FA spots were separately calculated using Imaris software (Bitplane). The number of focal adhesion spots was 235.7 ± 31.7 and the FA area of cells was 16 ± 4.2 μm^2^. Notably, vinculin expression on gelatin matrices was widely distributed throughout the entire cell-adhesion region. These results suggest that gelatin matrices provide a beneficial topography for preosteoblast spreading and FA assembly (Fig. 1l).

**Fig. 2:**
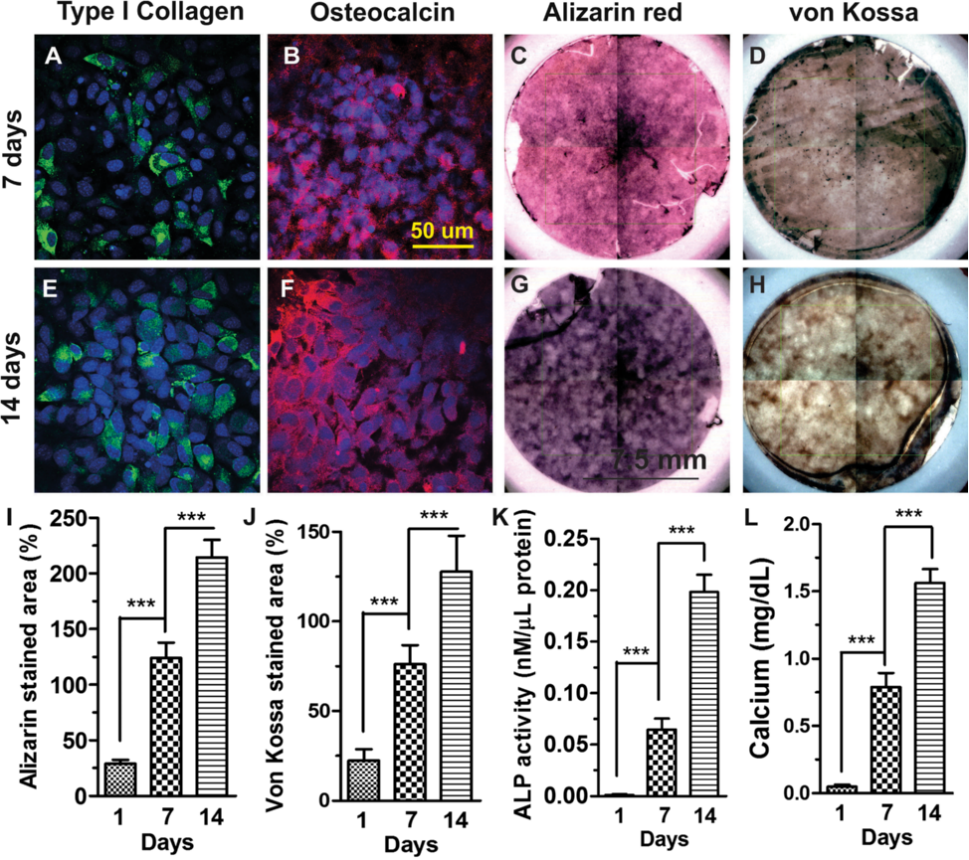
Osteogenic induction of preosteoblasts on gelatin matrices. Immunofluorescence images of **a**, **e** type I collagen and **b**, **f** osteocalcin at day 7 and 14, respectively. **c**, **g**) Alizarin red and **d**, **h** von Kossa staining of preosteoblasts at day 7 and 14, respectively. Quantification of **i** alizarin red, **j** von kossa positively-stained area, **k** normalized ALP activity, and **l** calcium content, respectively. Results are mean ± SD of one triplicate approach that is representative of three independent experiments. *** *p* < 0.001 indicates a statistically significant difference

The osteogenic potential of preosteoblasts on gelatin matrices was analyzed via von Kossa and alizarin staining, osteogenic gene expression, calcium content and ALP activity. Upon examining osteogenic proteins, osteocalcin (OC/green) and type I collagen (Col I/red) via immunofluorescence, we found stronger positive signals at day 7, which increased further by day 14 (Fig. 2a, b, e, f). The results demonstrated that the staining intensity significantly increased in a time-dependent manner. Meanwhile, qualitative analysis of mineralization via alizarin red (calcium) and von Kossa staining (phosphate) indicates an intense level of mineralization at day 7, which increased 2-fold by day 14 (Fig. 2c, d, g, h). Positively-stained areas of von kossa and alizarin red were quantitatively measured using Image J (Fig. 2i, j). Normalized ALP activity and calcium content were also found to increase significantly from day 1–14, indicating the osteogenic induction of preosteoblasts on gelatin matrices (Fig. 2k, l). Current results prove the positive impact of gelatin matrices towards the induction of the osteogenesis.

**Fig. 3:**
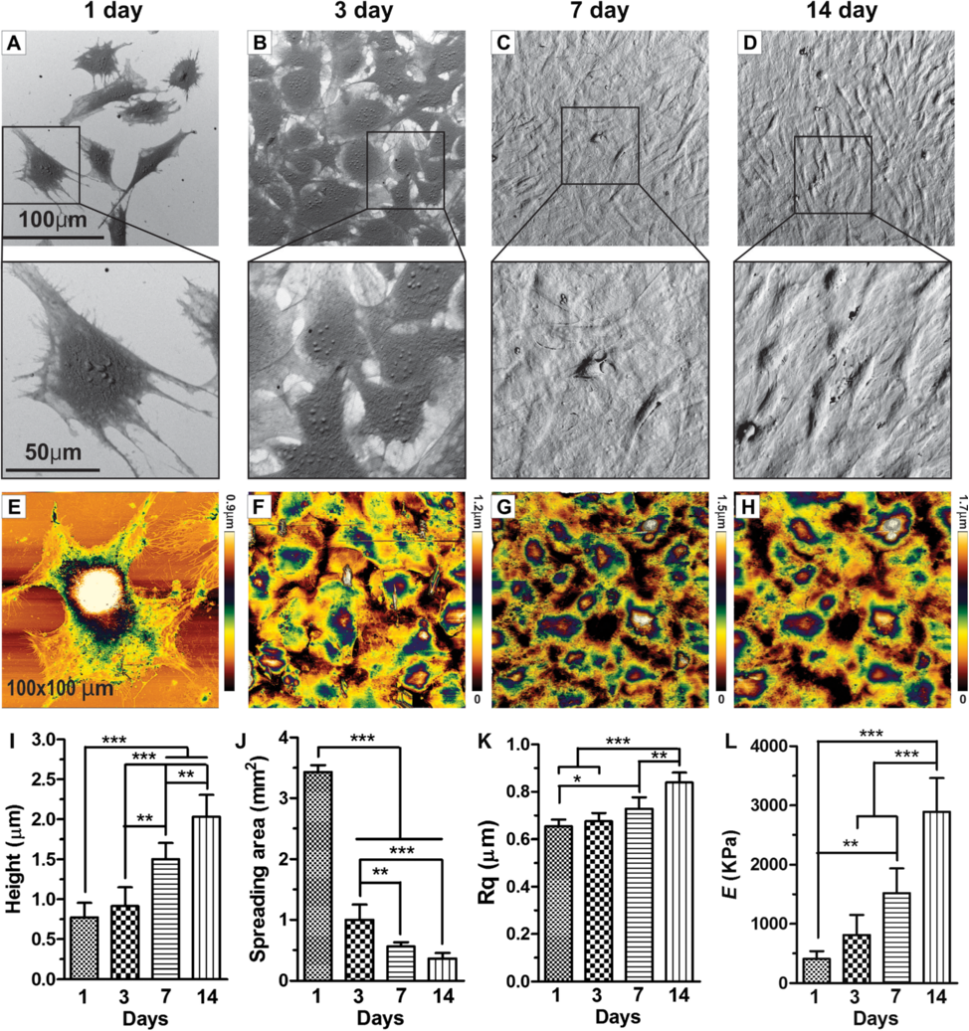
Morphology, biophysics and biomechanics of differentiating preosteoblasts. SEM **a**, **b**, **c**, **d** and AFM scanning **e**, **f**, **g**, **h** images of differentiating preosteoblasts on days 1, 3, 7, and 14 (100 × 100 μm scale). The obtained data were processed using JPK software. Quantitative data of **i** average cell height, **j** cell spreading area, **k** surface roughness, and **l** cell stiffness (*E*). Results are mean ± SD of one triplicate approach that is representative of three independent experiments. *** *p* < 0.001, ** *p* < 0.01, or * *p* < 0.05 is marked as a statistically significant difference

**Fig. 4:**
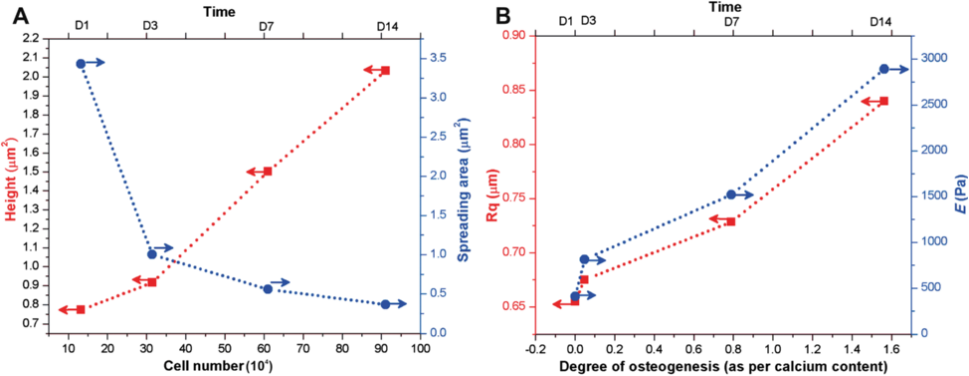
Correlation between osteogenesis and cellular properties. **a** Relationship of cell height and spreading area with respect to cell proliferation on days 1, 3, 7, and 14. **b** Integration of cell roughness and stiffness with respect to degree of osteogenic differentiation

Generally, biochemical markers have been used solely to analyze the differentiation of cells. However, physicomechanical properties during cell differentiation [11] can also provide new insights into cell behavior changes. In this study, we have demonstrated how biophysical (Rq) and biomechanical (*E*) factors can be developed as a biomarker for osteogenesis. AFM, SEM, and micro indentation-based analyzes were utilized to evaluate the structure, height, spreading area, roughness, and stiffness of preosteoblasts in a time-dependent manner. AFM-micro indentation analysis was made sequentially on days 1, 3, 7 and 14. Fig. 3 shows the SEM and AFM images of preosteoblasts at predetermined times; quantitative results were obtained using image processing software (JPK instruments and Image J, NIH). They clearly demonstrate that cells were widespread and experience strong filopodial interactions with the underlying gelatin matrices and neighboring cells at day 1. With time, these interactions gradually developed into a stronger interconnection between cells. The average height of preosteoblasts on days 1, 3, 7 and 14 was 0.8, 1.0, 1.5, and 2.1 um^2^, respectively. Significant changes in preosteoblast height were noticed from day 3. The cell height increased approximately 3 fold by day 14 (Figs. 3i and 4a). On the other hand, cell spreading area significantly declined from 3432 μm^2^ to 364 μm^2^ on gelatin matrices from day 1 to 14 (Figs. 3j & 4a). This drastic decline in spreading area is most likely due to increased cell proliferation (Fig. 4a). The results show that cell height is inversely proportional to spreading area. During the given time period, the roughness (Rq) of cell surfaces climbed sequentially from 655 nm to 840 nm (Figs. 3k and 4b). Furthermore, micro indentation analysis indicates that the average *E* (*n* = 50) for undifferentiated preosteoblasts on day 1 is 409 ± 130 Pa, and gradually increases to 813 ± 340 (Day 3), 1520 ± 416 (Day 7), and 2890 ± 570 Pa (Day 14). The overall cell stiffness after osteogenic differentiation increased 7 fold from day 1 to day 14 (Figs. 3l and 4b). These results evince that preosteoblasts undergo significant shifts in their phenotype behavior and physicomechanical properties during osteognic differentiation. We found that the progress of osteogenesis is directly proportional to the hike of cell stiffness and roughness. Height and spreading area are interplayed by cell proliferation rate, but exhibit no significant effect on osteogenesis. The significant changes in the cellular physicomechanical properties are due to mineralization of matrix and high protein secretion during osteogenic differentiation. Because, these specific changes were observed only for osteogenic differentiation, and different results are expected for different cell types. It is also important to note that AFM-based micro or nano indentation methods utilize various types of AFM tips and modes of operation which could result in a variance in the physicomechanical properties. Hence, an appropriate protocol should be implemented for AFM-indentation evaluation.

## Conclusions

We found out that gelatin matrices offer a favorable microenvironment for osteogenic differentiation of preosteoblasts in-vitro. Qualitative and quantitative assessments of osteogenesis show a time-dependent increase of the stiffness and roughness in the cellular and extracellular regions. The progress of osteogenesis is directly proportional to the roughness and stiffness of preosteoblasts while cell height and spreading area have little effect. Hence, stiffness and roughness of cells can possibly be understood as biomarkers.
