# Erratum to: Investigation of the changes of biophysical/mechanical characteristics of differentiating preosteoblasts in vitro

**DOI:** 10.1186/s40824-016-0052-8

**Published:** 2016-02-19

**Authors:** Ramesh Subbiah, Muhammad Suhaeri, Mintai Peter Hwang, Woojun Kim, Kwideok Park

**Affiliations:** Center for Biomaterials, Korea Institute of Science and Technology (KIST), Hwarangno 14-gil 5, Seongbuk-gu, Seoul, 136-791 Korea; Department of Biomedical Engineering, Korea University of Science and Technology (UST), Daejon, 305-350 Korea

In the original version of this article [1], there were three errors:

- In the description of the test for cell viability, “4 μM ethidium bromide” should be replaced by “4 μM ethidium homodimer-1 (EthD-1)”.
- In the Results and discussion section, the sentence “The surface morphology of gelatin matrix is visualized using AFM and SEM, respectively (Fig. 1b and c)” should read “The surface morphology of gelatin matrix is visualized using SEM and AFM, respectively (Fig. 1b and c).”
- In the Results and discussion section, the sentence “AFM images also depicted a relatively smooth gelatin surface with an RMS Rq of 155.6 ± 18 nm (Fig. 1d)” should read “AFM images also depicted a relatively smooth gelatin surface with an RMS Rq of 155.6 ± 12.3 nm (Fig. 1d)”.
